# Transcriptome data set of *Cellvibrio japonicus* grown using β-glucan media

**DOI:** 10.1128/mra.01491-25

**Published:** 2026-02-23

**Authors:** Jiabao Liang, Josephine J. Szczyrbak, Jessica K. Novak, Jeffrey G. Gardner

**Affiliations:** 1 Department of Biological Sciences, University of Maryland, Baltimore County 14701, Baltimore, Maryland, USA; California State University San Marcos, San Marcos, California, USA

**Keywords:** β-glucan, carbohydrate-active enzyme, *Cellvibrio japonicus*, polysaccharide

## Abstract

*Cellvibrio japonicus* is a gram-negative, saprophytic bacterium that is a polysaccharide utilization specialist capable of degrading diverse, complex, and recalcitrant polysaccharides. Here, we present transcriptome data sets during exponential growth and stationary phase for *C. japonicus* using barley β-glucan as the sole carbon source.

## ANNOUNCEMENT

*Cellvibrio japonicus* Ueda107 (NCIMB 10462) is a gram-negative, saprophytic bacterium isolated from Japanese field soil in 1948 (1, 2). Since its discovery, *C. japonicus* has been experimentally shown to utilize a wide variety of plant and animal polysaccharides as sole carbon sources (3, 4). Microbial polysaccharide degradation is of increasing interest due to its applications in biotechnology, most notably for renewable energy (5–7). Previous transcriptomic studies have revealed that many carbohydrate-active enzymes (CAZymes) are upregulated when *C. japonicus* is grown using recalcitrant polysaccharides as the sole carbon source (8–10). However, the gene expression profile of *C. japonicus* when grown using β-glucans has yet to be studied (11). Here we announce transcriptomic data sets for *C. japonicus* grown using barley β-glucan.

*C. japonicus* Ueda107 was obtained from the National Collections of Industrial and Marine Bacteria (Aberdeen, UK) as a lyophilized powder and revived using MOPS minimal media (TeKNova, cat. no. M2106) with 0.5% (wt/vol) glucose as the sole carbon source and solidified with 1.5% (wt/vol) agar. After a 48-hour incubation at 30°C, a single colony was inoculated into a 5 mL broth of MOPS minimal media with 0.5% (wt/vol) glucose and grown overnight at 30°C with high aeration (200 rpm), then 1 mL was stored in a −80°C freezer as a 50% (vol/vol) sterile glycerol stock. From the −80°C stock, a sample was sterilely streaked onto a MOPS-glucose plate and grown for 48 hours at 30°C. Single colonies were inoculated into three 5 mL broth cultures and incubated overnight at 30°C with high aeration (200 rpm) and subsequently used as the inoculum (1:100 dilution) for the RNAseq experiments, which replaced glucose with 0.5% (wt/vol) barley β-glucan (MegaZyme, cat. no. p-BGBM). The bacterium was grown at 30°C in 1 L shake flasks with 250 mL medium in biological triplicate with high aeration (200 rpm), and 35 mL samples were collected during mid-exponential (OD_600_ ~0.2) and stationary (OD_600_ ~1.0) phases for transcriptome analysis. After removal from the flasks, *C. japonicus* cells were treated with 5 mL of a stop solution composed of absolute ethanol (Sigma-Aldrich, cat. no. E7023) and acidic phenol (Sigma Aldrich, cat. no. P4557) (19:1; vol/vol ethanol:phenol ratio) and placed in a container of wet ice for 5 minutes to arrest cellular metabolism. Cells were next centrifuged at 8,000 × *g* for 5 minutes at 4°C, and the supernatants were discarded. Cell pellets were stored in a −80°C freezer before being shipped on dry ice to GeneWiz (South Plainfield, NJ, USA) for RNA extraction, cDNA library preparation, and sequencing.

Total RNA extraction was performed using the Qiagen RNeasy Mini Kit (Qiagen, cat. no. 74104) following the manufacturer’s instructions. The quality and integrity of total RNA samples were assessed using a Qubit 4.0 Fluorometer (Life Technologies, Carlsbad, CA, USA) and a 4200 TapeStation (Agilent Technologies, Palo Alto, CA, USA), respectively. Following rRNA depletion with a QIAseq FastSelect Kit (Qiagen cat. no. 335925), library preparation used a NEBNext Ultra II RNA Library Preparation Kit (NEB, cat. no. E7770S), with both being used as per the manufacturer’s recommendations. Libraries were multiplexed and clustered onto a flow cell on an Illumina HiSeq 2500 according to the manufacturer’s instructions using a 2 × 150 bp configuration. The raw sequence data generated from Illumina sequencing were output as bcl files. Using Illumina bcl2fastq software (v2.20), the bcl files were converted into FASTQ files and de-multiplexed. The FASTQ files were the deliverables from GeneWiz and submitted to NCBI SRA (Table 1).

**TABLE 1:** Quality overview of sequenced transcriptome

Sample identities	No. of reads^*a*^	Mean quality score	SRA accession no.
BAR^*b*^, EXP^*c*^, REP1^*d*^	19,363,278	35.90	SRX31360596
BAR, EXP, REP2	21,421,258	35.91	SRX31360597
BAR, EXP, REP3	19,442,472	35.89	SRX31360598
BAR, STA^*e*^, REP1	19,515,288	35.55	SRX31360599
BAR, STA, REP2	16,669,146	35.71	SRX31360600
BAR, STA, REP3	18,289,508	35.53	SRX31360601

^*a*^ Read length for all samples is 150 bp.

^*b*^ Barley β-glucan.

^*c*^ Exponential phase sample.

^*d*^ Replicate.

^*e*^ Stationary phase sample.

## Data Availability

The raw sequence data can be found under NCBI BioProject ID PRJNA1375792 and NCBI SRA IDs SRX31360596, SRX31360597, SRX31360598, SRX31360599, SRX31360600, and SRX31360601.
